# Prevalence and predictors of high-dose antipsychotic therapy among adult psychiatric inpatients in Baghdad, Iraq

**DOI:** 10.4102/sajpsychiatry.v32i0.2606

**Published:** 2026-01-14

**Authors:** Ola A. Nassr, Raghad F. Wadeea

**Affiliations:** 1 Department of Clinical Pharmacy, College of Pharmacy, Mustansiriyah University, Baghdad, Iraq

**Keywords:** high-dose antipsychotics, antipsychotic polypharmacy, psychosis, audit, antipsychotics, mental health

## Abstract

**Background:**

Current evidence-based guidelines recommend the use of antipsychotic monotherapy at the lowest effective dose. Nonetheless, high-dose antipsychotics and antipsychotic polypharmacy appear to be common in clinical practice, often deviating from established recommendations.

**Aim:**

This study aimed to estimate the prevalence and factors associated with high-dose antipsychotic prescribing among adult psychiatric inpatients.

**Setting:**

The study was conducted at Ibn Rushd Psychiatric Teaching Hospital in Baghdad, Iraq.

**Methods:**

The medical records of inpatients admitted from 24 April 2023 to 12 September 2023, were retrospectively analysed to extract routinely collected patient-level data and medication details; dosing appropriateness was based on that stated in the British National Formulary.

**Results:**

Of the 225 eligible patients, 51.1% were male, aged 18–82 years (mean = 33.9). Altogether, 48.6% of patients received antipsychotic polypharmacy, and 35.6% were prescribed high-dose antipsychotics. No significant associations were found between high-dose antipsychotic prescribing and patients’ characteristics, including age, sex, length of hospital stay, and number of admissions. Predictors of high-dose antipsychotics were polypharmacy (adjusted odd ratio [AOR]:12.61; 95% confidence interval [CI]: 1.78, 89.50), first-generation antipsychotics (AOR: 7.049; 95% CI 1.33, 37.44), quetiapine (AOR: 5.66; 95% CI 1.16, 27.53), procyclidine (AOR: 0.17; 95% CI 0.05, 0.55), and antidepressants (AOR: 0.19; 95% CI 0.05, 0.76).

**Conclusion:**

Approximately one in three patients received regular high-dose antipsychotic therapy, which contradicts optimal clinical practice and risks patient safety. Targeted educational interventions are warranted to enhance guideline adherence and promote safe and appropriate use of antipsychotics.

**Contribution:**

This is the first study to assess the magnitude and factors associated with high-dose antipsychotic prescription in Iraq.

## Introduction

Antipsychotic medications constitute a critical intervention for acute and long-term management of severe mental illness such as psychosis, bipolar disorder and treatment-resistant cases of major depressive disorder.^1^ However, these medications are associated with serious adverse effects; therefore, clinical practice guidelines recommend principles of rational prescribing and monitoring to enhance patient safety.^2,3^ Antipsychotic medications are broadly divided into two categories: first-generation antipsychotics (FGAs), which include haloperidol, chlorpromazine and sulpride, and second-generation antipsychotics (SGAs), which include olanzapine, quetiapine and risperidone.^1^ First-generation antipsychotics act through dopamine receptor (D2) antagonism in the brain, whereas SGAs block serotonin (5-HT2A) and dopamine (D2) neurotransmission.^1^

Evidence-based guidelines recommend antipsychotic monotherapy prescribed at the lowest effective dose.^2,3^ The use of high-dose antipsychotic therapy, whether originating from single or multiple medications, is not currently endorsed by guidelines.^2,3^ According to the American Psychiatric Association and the British Association for Psychopharmacology, high-dose antipsychotic therapy is reserved as the last resort for the management of treatment-resistant cases, which fail to achieve optimal response to an adequate trial of two antipsychotics from two different classes, and clozapine is tried and is either ineffective or not tolerated.^2,3^

Despite guidelines recommendations, observational studies show that prescription of antipsychotic polypharmacy and high-dose antipsychotics appear to be common in clinical practice.^4^ The prevalence of antipsychotic polypharmacy among inpatients varies globally between 20% and 66%.^4^ According to a study in Australia, the rate of high-dose antipsychotics is 27.0% among patients receiving compulsory treatment in the community.^5^ Similarly, a recent study in Italy reported a high rate of 50.8% among 730 forensic psychiatric inpatients.^6^ Furthermore, a study in Cyprus found that among 482 patients in psychiatric compulsory care settings, the rate of high-dose prescription was 27.2%.^7^ High-dose prescribing has been linked to various factors such as younger age, male gender, schizophrenia diagnosis, inpatient status, greater number of admissions, longer duration of hospital stay, prescription of antipsychotic polypharmacy, long-acting antipsychotics, anticholinergics, or FGAs.^8,9^

In acute adult psychiatric settings, antipsychotic polypharmacy, a key contributor to high-dose prescribing, is often used to stabilise patients with behavioural disturbances, promote rapid control of symptoms, and return the patient to baseline level of functioning.^10^ However, according to neuroimaging studies, increasing the dose does not necessarily increase the clinical response rates.^11^ Side effects of antipsychotics are dose-related.^12^ Therefore, increasing the dose increases the frequency and severity of side effects such as sedation, hypotension, extrapyramidal side effects, hyperprolactinaemia, neuroleptic malignant syndrome, seizure, arrhythmia, and sudden cardiac death.^12^ Thus, because of the potential harm associated with high-dose antipsychotic prescribing, physical and metabolic health monitoring has become a key performance indicator for patients receiving high-dose in the United Kingdom, North America, and Australia.^13^

Patients with severe mental illness exhibit a reduced life expectancy compared to the general population, which is attributed, in part, to the prescription of combined and high-dose antipsychotics.^9^ High-dose antipsychotics increase the risk of adverse drug events such that the incidence of sudden cardiac death is approximately doubled in patients receiving high doses compared to those receiving low doses.^8,14^ Furthermore, recent evidence indicates that antipsychotic dose reduction in patients with schizophrenia led to improvement in cognitive function without an increase in the risk of relapse.^15^ Evaluation of high-dose antipsychotic prescription trends can assess adherence to prescription guidelines and inform policymakers on implementing strategies to optimise treatment outcomes. Most of the evidence related to high-dose antipsychotic prescribing is derived from studies conducted in developed countries; studies evaluating the magnitude of high-dose antipsychotic prescribing in Iraq are currently lacking. We aim to address this gap in the literature by determining the prevalence and predictors of high-dose antipsychotic therapy among adult psychiatric inpatients in Baghdad, Iraq.

## Research methods and design

### Study design and setting

This study was an observational cross-sectional analysis of adult patients who received inpatient psychiatric care at the Ibn Rushd Psychiatric Teaching Hospital in Baghdad. This psychiatric hospital has delivered outpatient and inpatient mental health services since 1968 and is one of the two mental health hospitals in Iraq. The outpatient department is divided into four specialised units catering to distinct patient populations: children, adults, the elderly, and those with substance abuse issues, with an average of 150 patient consultations per working day. The inpatient department comprises six psychiatric wards with 48 beds and two substance abuse wards with 16 beds. The hospital employs an electronic database system, where each patient is assigned a unique healthcare number, enabling researchers to access information on inpatient admissions and outpatient visits using this identifier. Reporting of the study findings was guided by adhering to the Strengthening the Reporting of Observational Studies in Epidemiology (STROBE) guidelines.^16^

### Study population and data collection

Convenience sampling was employed to extract the information of patients receiving antipsychotic therapy during admission from 24 April 2023 to 12 September 2023. No sample size calculation was necessary because we included all patients admitted during the study period.

All adult patients with a diagnosis of schizophrenia and related psychotic disorders, mood disorders, or substance abuse receiving antipsychotic medication(s) were included. Patients under 18 years old, those with neurocognitive disorders such as Alzheimer’s disease, or personality disorders were excluded. The researcher reviewed 245 patient records; 20 were excluded because of age (*n* = 11), no antipsychotic medication prescribed (*n* = 4), neurological diseases (*n* = 4), and personality disorder (*n* = 1), leaving 225 patients eligible for inclusion. If there were multiple admissions during the study period, the first admission was considered. Given the exploratory nature of the study, teaching commitment of the first author, and resource implications, a sample size of 225 patients was deemed appropriate to represent the prescription pattern at the site.

The data collected included sociodemographic information such as age and sex, clinical information including psychiatric diagnosis and readmissions during the study period, and medication information such as medication name, total daily dose, and route of administration. This information was extracted from the electronic medical records by one experienced clinical pharmacist. The data were then entered into Excel, cleaned, and transferred to Statistical Package for the Social Sciences (SPSS) for analysis.

### Measurement of outcomes

The primary endpoint of the study was the rate of high-dose antipsychotic therapy. The British National Formulary (BNF) was consulted to determine the maximum doses for antipsychotics. The maximum doses of FGAs such as intramuscular (IM) chlorpromazine and IM haloperidol, and IM fluphenazine deconate depot were 200 mg/day, 20 mg/day, and 50 mg/week, respectively. The maximum daily doses for SGAs such as olanzapine, quetiapine, and risperidone were 20 mg, 800 mg, and 16 mg, respectively. For the purpose of the study, high-dose antipsychotic therapy was defined as the total daily dose of single agent being more than the maximum recommended dose, or the dosage where the sum of the percentages of the maximum doses of two or more antipsychotics received by the patient was more than 100%.^7,11^ For example, if the patient was prescribed quetiapine 400 mg, this would equate to 50% of the maximum recommended dose.

### Statistical analysis

Descriptive statistics were used to indicate patients’ characteristics and medication details. Data were represented as *n* (%) for categorical variables, and mean (standard deviation [s.d.]) or median (interquartile range [IQR]) for continuous variables. To test the association between categorical variables, the Pearson chi-squared test was used; for the continuous variables, the Mann–Whitney U test was used. Binary logistic regression using the enter method was used to identify the predictors of high-dose antipsychotic therapy. Data were analysed using SPSS version 22.0 (IBM Corp., Chicago, IL, US) and a *p*-value < 0.05 was used to indicate statistical significance.

### Ethical considerations

Ethical clearance to conduct this study was received from the institutional review boards of both the College of Pharmacy, Mustansiriyah University, and the Iraqi Ministry of Health (reference no: 130133). Data were deidentified to protect patient confidentiality.

## Results

### Socio-demographic and clinical characteristics of the study population

Of the 225 patients admitted during the study period, 115 (51.1%) were male and 110 (48.9%) were female. The median age was 30.0 years (range: 18–82), with a mean age of 33.9 (s.d. = 13.46). Most patients (79.1%) had a diagnosis of schizophrenia or psychosis, 15.6% had a diagnosis of mood disorders, and 5.3% had substance-related mental disorders. The median duration of hospital stay was 6 days, with a range of 1–41 days. Nearly 14% were readmitted during the study period, and 20.4% left against medical advice (Table 1).

**TABLE 1:** Demographic characteristics and frequency of prescribed medications (*N* = 225).

Characteristic	*N*	%	Mean	Standard deviation (s.d.)	Median	Range
Age, mean (s.d.), range (years)	-	-	33.9	13.46	-	18–82
Sex						
Male	115	51.1	-	-	-	-
Female	110	48.9	-	-	-	-
Psychiatric diagnosis						
F 10–19 (substance-related mental disorders)	12	5.3	-	-	-	-
F 20–29 (schizophrenia and psychosis)	178	79.1	-	-	-	-
F 30–40 (mood disorders)	35	15.6	-	-	-	-
Duration of hospital stay, median, range (days)	-	-	-	-	6	1–41
Multiple admissions during study period	32	14.2	-	-	-	-
Left against medical advice	46	20.4	-		-	-
Number of antipsychotics, median, (range)	-	-	-	-	1	1–3
One	118	52.4	-	-	-	-
Two	80	35.6	-	-	-	-
Three	27	12.0	-	-	-	-
Antipsychotic medication classes						
First-generation	52	23.1	-	-	-	-
Chlorpromazine	31	13.8	-	-	-	-
Haloperidol	22	9.8	-	-	-	-
Fluphenazine	6	2.7	-	-	-	-
Second-generation	225	100.0	-	-	-	-
Olanzapine	165	73.3	-	-	-	-
Quetiapine	125	56.1	-	-	-	-
Risperidone	9	4.0	-	-	-	-

### Prescription pattern of antipsychotics

Just over one half (52.4%, *n* = 118) of patients received antipsychotic monotherapy, while 47.6% (*n* = 107) were prescribed polypharmacy: 35.6% received two antipsychotics and 12.0% received three antipsychotics. All patients received SGAs, while only 23.1% of them also received FGAs. The most commonly prescribed medication was olanzapine (73.3%) followed by quetiapine (56.1%) and chlorpromazine (3.8%), while the least prescribed was fluphenazine depot injection (2.7%). Procyclidine, an anticholinergic medication, was given to 37.9%. Other psychotropic medications prescribed concomitantly included benzodiazepines (80.4%), mood stabilisers (75.1%), and antidepressants (23.6%).

All patient recipients of antipsychotic monotherapy were prescribed SGAs. For those receiving antipsychotic polypharmacy, the most commonly used combination was two agents from the SGAs, accounting for 43.0%, followed by one agent from FGAs and another agent from SGAs (30.8%). Nearly a quarter of patients (25.2%) received two agents from SGAs and one from the FGAs, and only one patient (0.9%) received two agents from FGAs and one from the SGAs. The most commonly used combination was olanzapine and quetiapine (41.1%), followed by olanzapine, quetiapine, and chlorpromazine (15.9%), and olanzapine and haloperidol (8.4%) (see Table 2).

**TABLE 2:** Prescription patterns of antipsychotic medications.

Characteristics	*N* = 225	
	*n*	%
**Monotherapy**	118	52.4
Olanzapine	72	61.0
Quetiapine	43	36.4
Risperidone	3	2.5
**Combination therapy**	107	47.6
SGA (2)	46	43.0
FGA (1) + SGA (1)	33	30.8
SGA (2) + FGA (1)	27	25.2
FGA (2) + SGA (1)	1	0.9
**Dual combination medications**	80/107	74.8
Olanzapine + quetiapine	44	41.1
Haloperidol + olanzapine	9	8.4
Chlorpromazine + olanzapine	7	6.5
Chlorpromazine + quetiapine	5	4.7
Fluphenazine + olanzapine	5	4.7
Haloperidol + quetiapine	3	2.8
Chlorpromazine + risperidone	3	2.8
Olanzapine + risperidone	3	2.8
Fluphenazine + quetiapine	1	0.9
**Triple combination medications**	27/107	25.2
Chlorpromazine, olanzapine, quetiapine	17	15.9
Haloperidol, olanzapine, quetiapine	8	7.5
Haloperidol, chlorpromazine, olanzapine	1	0.9
Haloperidol, olanzapine, risperidone	1	0.9

FGA, first-generation antipsychotics; SGA, second-generation antipsychotics.

### Prevalence and predictors of high-dose antipsychotics

In this study, 35.6% (*n* = 80) received high-dose antipsychotic therapy, which was through antipsychotic polypharmacy in 93.8% (*n* = 75) of cases, and only 6.2% (*n* = 5) had high-dose as a result of using one medication exclusively, namely, olanzapine at a dose exceeding the maximum recommended dose. Nearly half of high-dose recipients, that is, 53.8% (*n* = 43), were prescribed doses between 100% and 150% of the high-dose limit, 45.0% (*n* = 36) were between 150% – 200% and 1.3% (*n* = 1) received more than 200%. Dosage details of prescribed antipsychotic medications are displayed in Table 3.

**TABLE 3:** Dosage details of antipsychotic medications.

Medication	Minimum	Maximum	Mean	Standard deviation
Quetiapine	100.0	400.0	201.6	50.77
Chlorpromazine	25.0	150.0	83.1	28.42
Fluphenazine	25.0	25.0	25.0	-
Olanzapine	5.0	40.0	17.9	5.12
Haloperidol	5.0	15.0	8.6	2.75
Risperidone	2.0	4.0	3.6	0.88

In the bivariate analysis, prescription of high-dose was not associated with age, sex, hospital stay, or the admission and discharge status. On the other hand, high-dose was associated with psychiatric diagnosis, polypharmacy, FGAs, olanzapine and quetiapine, benzodiazepines, antidepressants, and procyclidine (see Table 4). According to the multivariate binary logistic regression, the predictors of high-dose in the descending order were polypharmacy (adjusted odd ratio [AOR]: 12.61; 95% confidence interval [CI] 1.78, 89.50), FGAs (AOR: 7.05; 95% CI 1.33, 37.44), quetiapine (AOR: 5.66; 95% CI 1.16, 27.53), procyclidine (AOR: 0.17; 95% CI 0.05, 0.55), and antidepressants (AOR: 0.19; 95% CI 0.05, 0.76) (see Table 5).

**TABLE 4:** Comparison of demographic and clinical characteristics between recipients of standard versus high-dose antipsychotics.

Characteristics	Receiving high-dose						*P*-value Χ² test
	Yes 80 (35.6)		No 145 (64.4)		Total 225 (100.0)		
	*n*	%	*n*	%	*n*	%	
Age, median (IQR)†	32.0	18.0	30.0	22.0	30.0	20.0	0.526
Sex	-	-	-	-	-	-	0.252
Male	35	15.6	75	33.3	110	48.9	-
Female	45	20.0	70	31.1	115	51.1	-
Hospital stay, median (IQR)†	7.5	9.8	6.0	4.8	6.0	6.8	0.135
Multiple admission	13	5.8	19	8.4	32	14.2	0.518
Left against medical advice	17	7.6	29	12.9	46	20.4	0.824
Psychiatric diagnosis	-	-	-	-	-	-	0.012
F 10–19	1	0.4	11	4.9	12	5.3	-
F 20–29	71	31.6	107	47.6	178	79.1	-
F 30–40	8	3.6	27	12.0	35	15.6	-
Polypharmacy	75	33.3	32	14.2	107	47.6	0.000
Antipsychotic medication	-	-	-	-	-	-	-
FGA	36	16.0	16	7.1	52	23.1	0.000
Olanzapine	80	35.6	85	37.8	165	73.3	0.000
Quetiapine	60	26.9	65	29.1	125	56.1	0.000
Concomitant medications	-	-	-	-	-	-	-
Benzodiazepines	74	32.9	107	47.6	181	80.4	0.001
Procyclidine	21	9.3	64	28.4	85	37.8	0.008
Antidepressants	6	2.7	47	20.9	53	23.6	0.000

Note: F 10–19: substance-related mental disorders; F 20–29: schizophrenia or psychosis; F 30–40: mood disorders.

FGA, first-generation antipsychotics; IQR, interquartile range.

† , Mann–Whitney *U* test was used to determine significance.

**TABLE 5:** Multivariate logistic regression analysis of factors independently associated with high-dose antipsychotic therapy.

Characteristics	Odds ratio	95% CI	*P-value*
Polypharmacy	12.61	1.78–89.49	0.011
FGA	7.05	1.33–37.44	0.022
Quetiapine	5.66	1.16–27.53	0.032
Procyclidine	0.17	0.05–0.55	0.003
Antidepressants	0.19	0.05–0.76	0.019

FGA, first-generation antipsychotics; CI, confidence interval.

## Discussion

In this study, all patients received SGAs, while 23.1% also received FGAs. Olanzapine was the most commonly prescribed antipsychotic medication at a rate of 73.3% followed by quetiapine at 56.1%. Polypharmacy was common, with 47.6% of patients receiving more than one antipsychotic medication: 35.6% received two antipsychotics and 12.0% received three. High-dose antipsychotics, which were prescribed to 35.6% of patients, result primarily from polypharmacy. Notably, 45.0% of high-dose prescriptions exceeded 150% of the recommended dose limit. Multivariate regression analysis indicated no association between high-dose antipsychotic prescription and demographic or clinical factors such as age, sex, psychiatric diagnosis, length of hospital stay, number of admissions, and discharge status. Predictors of receiving high-dose include antipsychotic polypharmacy, FGAs, quetiapine, antidepressants, and anticholinergic prescribing. Our findings are in line with an Australian study, which found that treatment-related factors were the primary predictors of high-dose antipsychotic prescribing.^5^

In this study, 35.6% received high-dose antipsychotic therapy. This rate is comparable to the 27.0% rate reported in Australia and Cyprus, but is lower than the 50.8% rate reported in Italy.^5,6,7^ There is no reliable evidence that increasing the dose beyond the standard dose is associated with better response; this practice is associated with increased frequency and severity of side effects, particularly sudden cardiac death.^17^ For antipsychotics to be effective, they should achieve 65% – 80% D2 receptor occupancy in the brain.^11^ Although there are reports of altered numbers or function of D2 receptors in a subgroup of patients, there is little evidence to support this, because therapeutic response is achieved in low to moderate doses.^11^ Schizophrenia and related psychoses are heterogeneous illnesses with multiple aetiologies, and in some patients, symptoms may not be dopamine-mediated. Therefore, increasing the dose of antipsychotics is clearly pointless.^11^ Furthermore, in a recent randomised study of 54 inpatients who were receiving high-dose antipsychotics before admission, lowering the administered antipsychotic dose to half, led to faster symptom improvement compared to those who stayed on high doses (1–3 weeks versus 4–9 weeks).^18^

Polypharmacy was a predictor of receiving high-dose, which is in line with other studies.^8^ In this study, only 6.2% received high-dose through monotherapy. A similar study in Italy indicates that patients on antipsychotic polypharmacy are 2.75 times more likely to receive high-dose antipsychotics.^6^ In the largest UK audit, the Prescribing Observatory for Mental Health, which analysed prescription patterns of antipsychotics among 3492 inpatients, found that 25.0% of inpatients were receiving high-dose antipsychotics, which occurred as a result of antipsychotic polypharmacy in 86.0% of cases.^19^ Similar findings were reported in a Saudi Arabian study.^20^

In this study, 47.6% of patients received polypharmacy which is similar to a study in Qatar that included inpatients and outpatients where 55.9% of patients were on monotherapy, 29.6% on dual and 14.5% on more than two agents.^21^ The antipsychotic polypharmacy rate was also similar to that reported in the United Kingdom (50.5%), Australia (43%), Canada (45.0%) and Italy (55.0%), but lower than 24.0% and 35.0% as reported in Japan and Denmark, respectively.^4,6,10,22,23,24^ As the number of prescribed antipsychotic increases, the life span shortens.^9^ Thus, prescribing antipsychotic polypharmacy is not only against guidelines recommendations but can also risk patient safety; even in treatment-resistant schizophrenia, clozapine monotherapy is recommended before antipsychotic polypharmacy.^2,3^ Evidence for antipsychotic polypharmacy is only available for clozapine in treatment-resistant schizophrenia. Clozapine has a weak D2 receptor affinity. The addition of potent D2 receptor blockers such as sulpride, amisulpride or aripiprazole, would provide a greater coverage of receptors for optimised antipsychotic efficacy.^25^ In addition, aripiprazole decreases the metabolic side effects of clozapine and thus, improves tolerability.^25^

Receiving FGAs was a predictor of receiving high-dose antipsychotics, which is in line with other studies.^26^ In this study, SGAs were the most commonly used, which is in line with a recent scoping review that indicates higher utilisation of olanzapine and quetiapine (SGAs) and lower prescription of haloperidol (FGAs).^12^ Second-generation antipsychotics are preferred by clinicians as first-line therapy; thus, the addition of FGAs to suppress residual positive symptoms may indicate treatment resistance.^10^ Thus, this may suggest that these patients may meet the criteria of treatment resistance and thus are more likely to receive high-dose. Likewise, SGAs were a predictor of receiving high-dose, which is in line with other studies.^26^ Quetiapine may also be employed as a hypnotic, and it also has antianxiety and antidepressant properties.^27^

Patients receiving high doses are less likely to receive procyclidine. Other studies reported the opposite, with high-dose antipsychotics increasing the likelihood of receiving anticholinergics.^8,28^ This may be because of our exclusion of prescribed as-required medications, which are haloperidol and chlorpromazine (FGAs). By including only regular medication, which are mostly SGAs, the need for anticholinergics is greatly reduced in line with other studies.^23^ Other studies also reported FGA use as a predictor of anticholinergic prescription.^28^ The APA advocates short-term prophylactic use of anticholinergics only in high-risk patients susceptible to dystonia, such as young males receiving parenteral highly potent FGA.^2^

Less antidepressant use was a predictor of high-dose, which is in line with other studies.^29^ Patients receiving high-dose have more severe psychopathology and are likely treatment-resistant; thus, antipsychotics rather than antidepressants constitute the primary treatment option for this group of patients.^11^ In addition, clinicians may be concerned about increasing the side effect burden and drug interactions emerging from the addition of antidepressants to high-dose antipsychotics.

In this study, no patients received clozapine, which aligns with the findings from a study in Qatar, where only 2.4% received clozapine.^30^ Clozapine is indicated in treatment-resistant schizophrenia, which affects 30.0% of patients with schizophrenia.^10^ However, this medication has a special protocol for initiation and titration and is associated with a list of side effects that require regular monitoring, such as agranulocytosis.^31^ Thus, acutely unwell patients may not consent to blood sampling, and this hinders its widespread use.^32^ In those with treatment resistance, only 4.0% trialled clozapine before using antipsychotic polypharmacy.^9^ This implies that polypharmacy is considered early and that clinicians may delay prescribing clozapine, which contributes to increased morbidity and mortality.^9^

This is the first national study to describe the magnitude and factors associated with high-dose antipsychotic therapy, providing a baseline for future monitoring of prescribing trends. The study is considered representative because the study site is one of two hospitals in Iraq that provide inpatient psychiatric care. Although the study design is retrospective, the data were collected by one experienced clinical pharmacist researcher, thus minimising variation and enhancing the data quality. However, because of the cross-sectional nature, causality cannot be established. In addition, we excluded as-required antipsychotic medications; thus, the rate of antipsychotic polypharmacy and high-dose antipsychotics may be underestimated.

We observed a high rate of antipsychotic polypharmacy and high-dose antipsychotics, which are unaligned with standard practice guidelines, increase side effects and drug interactions, and thus risk patient safety.^2,3^ Although antipsychotic polypharmacy may be required initially during acute hospitalisation, reverting to monotherapy is recommended long-term to sustain response.^12^ Recent evidence suggests that antipsychotic polypharmacy prescribing at discharge increases the risk of psychiatric readmission.^33^ Thus, the goal is to review treatment at discharge to ensure optimal evidence-based prescribing.

It is possible that clinicians were unaware that they were pursuing high-dose therapy because, in most cases, high-dose antipsychotics result from polypharmacy. Education programmes for clinicians are vital to enhance adherence to guidelines and ensure optimal delivery of psychopharmacological therapy. Clozapine and long-acting injectable antipsychotics are effective in reducing the risk of relapse, and thus, improving the utilisation of these agents as maintenance therapy, when appropriate, especially in those with multiple admissions, should be considered.^2,11^ Information regarding the adverse effects of antipsychotics or previous clozapine trial for those receiving antipsychotic polypharmacy is lacking in the medical records. Reasons for prescribing antipsychotic polypharmacy should be clearly documented in the medical records. Furthermore, the inclusion of pharmacists specialised in mental health as a part of a multidisciplinary team is important to enhance guideline adherence and promote optimal use of antipsychotics. Finally, a qualitative survey to assess the psychiatrists’ knowledge, attitudes, and practices towards the prescription of combined and high-dose antipsychotics is warranted.

## Conclusion

The study’s findings indicate that one in three patients received high-dose antipsychotics. Patients receiving combined antipsychotics or FGAs are particularly at risk. This practice, which is not endorsed by guidelines, should be reviewed to ensure patient safety. Targeted educational interventions are recommended to enhance adherence to prescription guidelines and optimise treatment outcomes.

## References

[1] Leucht S, Priller J, Davis JM. Antipsychotic drugs: A concise review of history, classification, indications, mechanism, efficacy, side effects, dosing, and clinical application. Am J Psychiatry. 2024;181(10):865–878. 10.1176/appi.ajp.20240738 39350614

[2] Keepers GA, Fochtmann LJ, Anzia JM, et al. The American Psychiatric Association practice guideline for the treatment of patients with schizophrenia. Am J Psychiatry. 2020;177(9):868–872. 10.1176/appi.ajp.2020.177901 32867516

[3] Barnes TR, Drake R, Paton C, et al. Evidence-based guidelines for the pharmacological treatment of schizophrenia: Updated recommendations from the British Association for Psychopharmacology. J Psychopharmacol. 2020;34(1):3–78. 10.1177/0269881119889296 31829775

[4] Ayani N, Morimoto T, Sakuma M, Kikuchi T, Watanabe K, Narumoto J. Antipsychotic polypharmacy is associated with adverse drug events in psychiatric inpatients. J Clin Psychopharmacol. 2021;41(4):397–402. 10.1097/JCP.0000000000001416 34108429 PMC8244930

[5] Gisev N, Bell JS, Chen TF. Factors associated with antipsychotic polypharmacy and high-dose antipsychotics among individuals receiving compulsory treatment in the community. J Clin Psychopharmacol. 2014;34(3):307–312. 10.1097/JCP.0000000000000098 24717256

[6] Mandarelli G, Carabellese F, Di Sciascio G, Catanesi R. Antipsychotic polypharmacy and high-dose antipsychotic regimens in the Residential Italian Forensic Psychiatric Population (REMS). Front Psychol. 2022;13:722985. 10.3389/fpsyg.2022.722985 35222172 PMC8866699

[7] Kaikoushi K, Karanikola M, Middleton N, Bella E, Chatzittofis A. Prescription patterns in psychiatric compulsory care: Polypharmacy and high-dose antipsychotics. BJPsych Open. 2021;7(5):e149. 10.1192/bjo.2021.982 34747353 PMC8388008

[8] Anozie IG, James BO, Omoaregba JO, Oriji SO, Erohubie PO, Enebe AC. Correlates of high-dose antipsychotic prescription amongst outpatients with Schizophrenia in a Nigerian Hospital. S Afr J Psychiatr. 2022;28:1791. 10.4102/sajpsychiatry.v28i0.1791 35547105 PMC9082254

[9] Langan J, Shajahan P. Antipsychotic polypharmacy: Review of mechanisms, mortality and management. Psychiatrist. 2010;34(2):58–62. 10.1192/pb.bp.108.024257

[10] Lassen S, Heintz T, Pedersen T, et al. Nationwide study on antipsychotic polypharmacy among forensic psychiatric patients. Int J Circumpolar Health. 2023;82(1):2218654. 10.1080/22423982.2023.2218654 37300837 PMC10259293

[11] Royal College of Psychiatrists. The risks and benefits of high-dose antipsychotic medication [homepage on the Internet]. College Report 190. 2024 [cited Apr 2025]. Available from: https://www.rcpsych.ac.uk/docs/default-source/improving-care/better-mh-policy/college-reports/college-report-cr190.pdf?sfvrsn=54f5d9a2_2

[12] Ying J, Chew QH, Wang Y, Sim K. Global neuropsychopharmacological prescription trends in adults with schizophrenia, clinical correlates and implications for practice: A scoping review. Brain Sci. 2023;14(1):6. 10.3390/brainsci14010006 38275511 PMC10813099

[13] Johnson CF, Ingram F, Thomson F, Srireddy P, Jani BD, Greenlaw N. General practice pharmacist-led antipsychotic physical health monitoring: A prospective intervention scoping study. Fam Pract. 2024;41(1):41–49. 10.1093/fampra/cmad120 38180874

[14] Ray WA, Chung CP, Murray KT, Hall K, Stein CM. Atypical antipsychotic drugs and the risk of sudden cardiac death. N Engl J Med. 2009;360(3):225–235. 10.1056/NEJMoa0806994 19144938 PMC2713724

[15] Singh A, Kumar V, Pathak H, et al. Effect of antipsychotic dose reduction on cognitive function in schizophrenia. Psychiatry Res. 2022;308:114383. 10.1016/j.psychres.2021.114383 34999291

[16] Cuschieri S. The STROBE guidelines. Saudi J Anaesth. 2019;13(Suppl. 1):S31–S34. 10.4103/sja.SJA_543_18 30930717 PMC6398292

[17] Samara MT, Klupp E, Helfer B, Rothe PH, Schneider-Thoma J, Leucht S. Increasing antipsychotic dose for non response in schizophrenia. Cochrane Database Syst Rev. 2018;5(5):CD011883. 10.1002/14651858.CD011883.pub2 29750432 PMC6494602

[18] Ataniya R, Koike T, Inamoto A. Halved dose of antipsychotics versus high-dose antipsychotic therapy for relapse in patients with schizophrenia receiving high-dose antipsychotic therapy: A randomized single-blind trial. Int J Mol Sci. 2025;26(9):4003. 10.3390/ijms26094003 40362244 PMC12071691

[19] Paton C, Barnes TRE, Cavanagh M-R, Taylor D, Lelliott P, POMH-UK Project Team. High-dose and combination antipsychotic prescribing in acute adult wards in the UK: The challenges posed by p.r.n. prescribing. Br J Psychiatry. 2008;192(6):435–439. 10.1192/bjp.bp.107.042895 18515893

[20] Alsabhan JF, Almalag HM, Aljafali L, Alnughamish H, Almutlaq G. Prescribing pattern of antipsychotics for patients with schizophrenia using the total daily dose online tool. Saudi Pharm J. 2023;31(12):101837. 10.1016/j.jsps.2023.101837 38033746 PMC10682108

[21] Aburamadan HAR, Sridhar SB, Tadross TM, Shariff A. Assessment of prescription pattern of antipsychotic medications in a psychiatry inpatient setting of a secondary care hospital of United Arab Emirates. Clin Schizophr Relat Psychoses. 2021;15(5):S8. 10.3371/CSRP.AHSS.073021

[22] Lelliott P, Paton C, Harrington M, Konsolaki M, Sensky T, Okocha C. The influence of patient variables on polypharmacy and combined high dose of antipsychotic drugs prescribed for in-patients. Psychiatric Bull. 2002;26(11):411–414. 10.1192/pb.26.11.411

[23] John AP, Gee T, Alexander S, Ramankutty P, Dragovic M. Prevalence and nature of antipsychotic polypharmacy among inpatients with schizophrenia spectrum disorders at an Australian mental health service. Aust Psychiatry. 2014;22(6):546–550. 10.1177/1039856214546672 25147317

[24] Farrell C, Brink J. The prevalence and factors associated with antipsychotic polypharmacy in a forensic psychiatric sample. Front Psychiatry. 2020;11:263. 10.3389/fpsyt.2020.00263 32528318 PMC7247840

[25] Lerner V, Libov I, Kotler M, Strous RD. Combination of ‘atypical’ antipsychotic medication in the management of treatment-resistant schizophrenia and schizoaffective disorder. Prog Neuropsychopharmacol Biol Psychiatry. 2004;28(1):89–98. 10.1016/j.pnpbp.2003.09.024 14687862

[26] Dong M, Zeng L-N, Zhang Q, et al. Prescription of antipsychotic and concomitant medications for adult Asian schizophrenia patients: Findings of the 2016 Research on Asian Psychotropic Prescription Patterns (REAP) survey. Asian J Psychiatr. 2019;45:74–80. 10.1016/j.ajp.2019.08.010 31520884

[27] Gaviria AM, Franco JG, Aguado V, et al. A non-interventional naturalistic study of the prescription patterns of antipsychotics in patients with schizophrenia from the Spanish Province of Tarragona. PLoS One. 2015;10(10):e0139403. 10.1371/journal.pone.0139403 26427051 PMC4591292

[28] Hori H, Yasui-Furukori N, Hasegawa N, et al. Prescription of anticholinergic drugs in patients with schizophrenia: Analysis of antipsychotic prescription patterns and hospital characteristics. Front Psychiatry. 2022;13:823826. 10.3389/fpsyt.2022.823826 35656353 PMC9152135

[29] Takahashi T, Otsubo T, Kunisawa S, Sasaki N, Imanaka Y. Factors associated with high-dose antipsychotic prescriptions in outpatients with schizophrenia: An analysis of claims data from a Japanese prefecture. Neuropsychopharmacol Rep. 2020;40(3):224–231. 10.1002/npr2.12109 32452649 PMC7722669

[30] Ouanes S, Becetti I, Ghuloum S, et al. Patterns of prescription of antipsychotics in Qatar. PLoS One 2020;15(11):e0241986. 10.1371/journal.pone.0241986 33166337 PMC7652328

[31] Bitter I, Dossenbach MRK, Brook S, et al. Olanzapine versus clozapine in treatment-resistant or treatment-intolerant schizophrenia. Prog Neuropsychopharmacol Biol Psychiatry. 2004;28(1):173–180. 10.1016/j.pnpbp.2003.09.033 14687871

[32] Gobbi G, Comai S, Debonnel G. Effects of quetiapine and olanzapine in patients with psychosis and violent behavior: A pilot randomized, open-label, comparative study. Neuropsychiatr Dis Treat. 2014;10:757–765. 10.2147/NDT.S59968 24855361 PMC4019623

[33] Kadra G, Stewart R, Shetty H, et al. Antipsychotic polypharmacy prescribing and risk of hospital readmission. Psychopharmacology. 2018;235(1):281–289. 10.1007/s00213-017-4767-6 29080904 PMC5748404

